# Coexistent ARID1A-PIK3CA mutations are associated with immune-related pathways in luminal breast cancer

**DOI:** 10.1038/s41598-023-48002-x

**Published:** 2023-11-27

**Authors:** Liat Anabel Sinberger, Tamar Zahavi, Amir Sonnenblick, Mali Salmon‐Divon

**Affiliations:** 1 Department of Molecular Biology, Ariel University, Ariel, Israel; 2 Institute of Oncology, Tel Aviv Sourasky Medical Center, Tel Aviv, Israel; 3 Sackler Faculty of Medicine, Tel Aviv University, Tel Aviv, Israel; 4 Adelson School of Medicine, Ariel University, Ariel, Israel

**Keywords:** Computational biology and bioinformatics, Genomics, Cancer, Breast cancer

## Abstract

Up to 40% of luminal breast cancer patients carry activating mutations in the PIK3CA gene. PIK3CA mutations commonly co-occur with other mutations, but the implication of this co-occurrence may vary according to the specific genes involved. Here, we characterized a subgroup of luminal breast cancer expressing co-mutations in ARID1A and PIK3CA genes and identified their effect on important signaling pathways. Our study included 2609 primary breast cancer samples from the TCGA and METABRIC datasets that were classified based on tumor subtype and the existence of mutations in PIK3CA and ARID1A genes. Differential expression and WGCNA analyses were performed to detect molecular modules affected by the existence of the mutations. Our results reveal various evidence for the involvement of immune-related pathways in luminal tumors harboring ARID1A and PIK3CA mutations, as well as a unique Tumor-infiltrated immune cells composition. We also identified seven key hub genes in the ARID1A-PIK3CA mutated tumors associated with immune-related pathways: CTLA4, PRF1, LCK, CD3E, CD247, ZAP70, and LCP2. Collectively, these results indicate an immune system function that may contribute to tumor survival. Our data induced a hypothesis that ARID1A and PIK3CA mutations' co-occurrence might predict responses to immunotherapy in luminal BC and, if validated, could guide immunotherapy development.

## Introduction

Breast cancer (BC) is the leading diagnosed cancer in females worldwide^1^. 60–70% of BC patients are diagnosed with luminal A and luminal B subtypes. These tumors are characterized by the presence of hormonal receptors, estrogen receptor (ER) and progesterone receptor (PR), and the absence of HER2 receptors on the cells (ER-positive/PR-positive)^2^. Luminal tumors are commonly treated with hormone therapy since ER plays a critical role in regulating their proliferation and endocrine response. However, despite the effectiveness of adjuvant endocrine therapies, metastatic patients often develop resistance to these treatments. Therefore, a more comprehensive understanding of the mechanisms involved in the various aspects of luminal cancers is necessary^3,4^.

Luminal breast cancers are often composed of cells with diverse genetic signatures, which can impact cancer progression and the ability to resist treatment. PIK3CA mutations resulting in the hyperactivation of the Phosphatidylinositol-3 kinase (PI3K) signaling pathway are present in approximately 40% of luminal cases. This pathway is crucial in various cellular processes, including cell proliferation, survival, and DNA repair. Therefore, targeting this pathway has become a therapeutic strategy for treatment of luminal tumors^5–7^. PI3Ks are heterodimeric lipid kinase enzymes consisting of regulatory (p85) and catalytic (p110) subunits, grouped into three different classes (I – III). PI3K signal is activated following the interaction of phosphotyrosine residues of activated growth factor receptors or adaptor proteins (e.g., RAS proteins) with the catalytic subunit of PI3Ks. Consequently, phosphatidylinositol-3,4,5-trisphosphate activates Akt, inducing multiple downstream effectors^6,7^. Mutations in the PIK3CA alpha isoform, which encodes for p110α, are frequently associated with primary luminal tumors^5^.

Additional mutations, such as in SWI/SNF chromatin remodeling complex subunit genes, are frequently observed in luminal tumors, with the ARID1A gene being the most commonly mutated target^8^. The SWI/SNF complex is essential for maintaining epigenetic modifications in an ATP-dependent manner^9^. It binds to enhancer or promoter regions of the DNA to regulate gene transcription in various cellular pathways and is also involved in DNA repair. An impaired function of this complex can result in genomic instability^10–12^. The ARID family proteins enhance the activity of the SWI/SNF complex by recruiting it to chromatin^13^. Inactivating mutations in ARID1A frequently occur in endocrine-resistant metastatic luminal cancer^4^. Mutations with clinical significance are more frequent in metastatic BC than in primary tumors^14^. For example, Razavi et al. showed that ARID1A loss-of-function mutations did not occur in primary tumors but, rather, were acquired in metastasis after treating luminal patients with endocrine therapy^15^.

Generally, cancer gene mutations do not occur randomly. Moreover, mutations of certain cancer genes tend to co-occur, suggesting they contribute together to tumor formation and development. Specifically, PIK3CA mutations commonly co-occur with other BC mutations, with varying implications, depending on the specific genes involved. The co-occurrence of ARID1A and PIK3CA mutations has been demonstrated in different cancer types. For example, in ovarian clear-cell carcinoma, which has a poor prognosis, ARID1A mutations are found in 50% of the tumors, and double mutations in ARID1A and PIK3CA were found in 33% of the tumors. Chandler et al. found that ovary tumors harboring the double mutation express genes involved in Interleukin-6 signaling, immune system function, and other pathways compared to normal ovary cells^12^. ARID1A-PIK3CA mutational co-occurrence was also demonstrated in gastric cancer^16^.

Here, we sought to characterize a new subgroup of luminal BC expressing co-mutations in ARID1A and PIK3CA genes. To accomplish this goal, we examined the frequencies and possible involvement of ARID1A and PIK3CA mutations' co-occurrence in the pathogenesis of luminal tumors.

## Materials and methods

### Data resource and study design

Normalized gene expression profiles, probe annotation and clinical information of 3054 samples were downloaded from the TCGA^17,18^ (1073 samples) and METABRIC^19–21^ (1,981 samples) databases using the MetaGxBreast R package^22^ (version 1.12.0). In addition to the clinical information obtained from MetaGxBreast, PAM50 classification (2,695 samples), information regarding somatic non-synonymous mutations (2,914 samples), tumor mutational burden (TMB) (2,606 samples), and protein expression measured by reverse-phase protein array (RPPA) z-score for TCGA samples (731 samples) were downloaded from cBioPortal for Cancer Genomics^23,24^. Samples without mutational or PAM50 information were excluded from the analysis, leaving 2,609 BC primary tumor samples. Throughout the analysis, we grouped luminal A and luminal B samples together (“luminal samples”). The classification was done according to PAM50.

**Figure 1:**
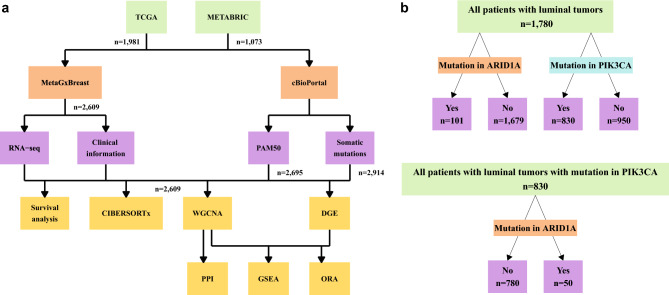
Study design and datasets. (**a**) Study design and (**b**) patients’ cohorts used for analysis.

Data processing was performed using R studio software (version 4.1.1)^25^. The study design and the comparisons performed are presented in Fig. 1a,b. The control group in this study consisted of tumor samples with no mutations in either ARID1A or the PIK3CA genes.

### Identification of differentially expressed genes (DEGs)

The limma^26^ (version 3.48.3) and edgeR^27,28^ (version 3.34.1) R packages were used for batch effect correction and detection of DEGs among the different groups. Unless specified otherwise, a false discovery rate (FDR) of < 0.05 and fold change > 1.5 were used as the statistical cutoff for differential expression.

### Weighted correlation network analysis (WGCNA)

The WGCNA^29,30^ R package (version. 1.70.3) was used to perform signed network analysis from all gene expression profiles for the different cohorts. For network construction, the selected power (β) was 12, and scale-free topology was set to a minimum of 0.8. Cluster dendrogram visualized the modules represented in different colors, and dynamic modules with high similarities were combined into one at a cutline of 0.25. Correlation between modules and different traits was examined to identify the correlative modules. Module membership (MM) and gene significance (GS) were generated for selected modules.

### Pathways enrichment analysis

Pathways enrichment analysis was performed against the Kyoto Encyclopedia of Genes and Genomes (KEGG) pathways database through the WebGestalt 2019^31^ tool. DEGs found in each comparison and genes included in WGCNA selected modules were used as input for over-representation analysis (ORA). Ranked gene lists with their corresponding logFC from each comparison were used as input for gene set enrichment analysis (GSEA). Only ontologies and pathways with FDR < 0.05 are shown.

### Identifying Hub genes

Hub genes are genes in candidate modules highly correlated to a specific trait in the WGCNA analysis. Unless otherwise mentioned, the top 10% of genes in a candidate module with MM > 0.8 and GS > 0.2 were considered hub genes^32^. These genes were used as input to the Search Tool for Retrieval of Interacting Genes/Proteins (STRING)^33^ database to perform protein–protein interaction (PPI) analysis. Visualization was performed using the Cytoscape softwere^34,35^. Genes with the highest degrees of connectivity in the PPI network play a critical role in the module^32^ and were therefore defined as key hub genes.

### Cell-type identification by estimating relative subsets of RNA transcripts (CIBERSORT)

Tumor-infiltrated immune cells were estimated using CIBERSORTx^36,37^. The gene expression profile was used as input. The proportion of twenty-two immune cell subtypes was measured in the absolute mode for each sample using an LM22 signature with 100 permutations for *p* value calculation. In WGCNA analysis, the abundance of each immune cell was treated as a continuous variable.

### Tumor infiltrated immune cells and gene expression correlation

The Pearson correlation between gene expression and tumor infiltrated immune cells absolute score detected by CIBERSORTx and the correlation significance were calculated using the corrplot^38^ R package (Version 0.92).

### Statistical analysis

This study's statistical analyses were conducted using R 4.1.1 statistical framework. Differences in categorical variables between groups were analyzed using Fisher's exact test. The nonparametric Kruskal–Wallis test followed by the Dunn post hoc test were performed to compare continuous parameters between different groups. Survival curves were estimated using the Kaplan–Meier method and visualized by Survival ^39,40^ (Version 3.5–3) and Survminer (Version 0.4.9) R packages, and *p* values were calculated using the log-rank test. Effects were considered statistically significant at a two-sided *p* < 0.05. All statistical analyses were performed using the R statistical framework (v.4.4.1). A ggplot2^41^ R package (Version 3.3.5) was used for generating plots. Venn diagrams were prepared using the VennDiagram^42^ (Version 1.7.3) R package.

## Results

### Coexistent ARID1A-PIK3CA mutations in luminal breast cancer

**Figure 2:**
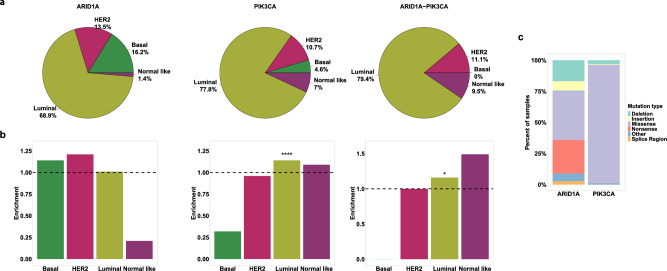
ARID1A-PIK3CA mutational co-occurrence enrichment in luminal tumors. (**a**) Pie charts depicting the distribution of ARID1A or PIK3CA-only mutations and coexistent ARID1A-PIK3CA mutations (in percentages) across breast cancer subtypes. The number of participants in each group are listed in Table 1. (**b**) Enrichment of ARID1A or PIK3CA-only mutations and coexistent ARID1A-PIK3CA mutations in breast cancer subtypes (*p ≤ 0.05, **** ≤ 0.0001, Fisher’s exact test). (**c**) Bar plot summarizing the distribution of ARID1A (n = 148) or PIK3CA (n = 1164) mutation types in breast tumors.

To assess the prevalence of ARID1A and PIK3CA mutations co-occurring in BC, we examined their frequency in various BC subtypes using the TCGA^17,18^ and METABRIC^19–21^ datasets, which included 2,609 patients. Of these patients 1,780 were classified as Luminal (including 1,134 Luminal A and 646 Luminal B patients), 291 were Her2-enriched, 371 were Basal and 167 Normal subtype. Most patients were over 50 years old, had tumor sizes ranging from 2.1 to 5 mm and exhibited tumor grades of 2–3. Among the patients with luminal tumors, 51 (2.86%) had mutations in ARID1A gene, 780 (43.82%) had mutations in PIK3CA gene, 50 (2.8%) patients had mutations in both the ARID1A and PIK3CA genes, and 899 (50.5%) women served as a control group showing no mutations in any of these genes (Table 1 and Fig. 1a,b). Our analysis revealed that PIK3CA mutations, as well as ARID1A-PIK3CA mutational co-occurrence, were significantly more common (*p* ≤ 0.05) in luminal tumors than in other BC subtypes (Fig. 2a,b). The distribution of ARID1A and PIK3CA mutation types in breast tumors is illustrated in Fig. 2c.

**Table 1:** Clinical features of the study samples.

PAM50 subtype	ARID1A mutation	PIK3CA mutation	ARID1A and PIK3CA mutations	Control (No mutations in ARID1A or PIK3CA)	PAM50 subtype
**Age**					
21–50					
Basal	137 (36.9%)	5	11	0	121
Her2	66 (22.68%)	2	19	1	44
Normal	56 (33.5%)	0	23	2	31
Lum	340 (19.1%)	14	145	12	169
*p* value*		0.3804	9.6e-09	0.1103	
51–97					
Basal	234 (63.1%)	7	35	0	192
Her2	225 (77.32%)	8	88	6	123
Normal	111 (66.5%)	1	47	4	59
Lum	1440 (80.9%)	37	635	38	730
*p* value*		0.7587	3.1e-09	0.3301	
**Tumor size (mm)**					
0–2.0					
Basal	101 (27.22%)	4	13	0	84
Her2	87 (29.9%)	4	28	2	53
Normal	69 (41.32%)	1	25	2	41
Lum	688 (38.65%)	18	331	20	319
*p* value*		0.5108	3.7e-10	0.3521	
2.1–5.0					
Basal	229 (61.73%)	7	28	0	194
Her2	172 (59.11%)	6	71	4	91
Normal	77 (46.11%)	0	37	3	37
Lum	946 (53.15%)	27	393	26	500
*p* value*		1	1.4e-06	0.1399	
> 5					
Basal	32 (8.63%)	1	3	0	28
Her2	23 (7.9%)	0	6	1	16
Normal	17 (10.18%)	0	5	1	11
Lum	114 (6.4%)	4	43	4	63
*p* value*		0.6502	0.0092	1	
**Grade**					
1					
Basal	1 (0.27%)	0	1	0	0
Her2	4 (1.37%)	1	2	0	1
Normal	11 (6.59%)	0	8	1	2
Lum	135 (7.58%)	1	94	5	35
*p* value*		0.2013	1	0.4955	
2					
Basal	16 (4.31%)	0	7	0	9
Her2	48 (16.49%)	2	17	2	27
Normal	71 (42.51%)	0	34	2	35
Lum	532 (29.89%)	15	249	13	255
*p* value*		0.5458	0.4406	0.7597	
3					
Basal	179 (48.25%)	7	24	0	148
Her2	152 (52.23%)	5	63	2	82
Normal	44 (26.35%)	0	18	3	23
Lum	410 (23.03%)	14	157	13	226
*p* value*		1	0.0024	0.09838	

*p value represents enrichment in luminal tumors versus the rest as calculated using Fisher exact test.

The PIK3CA gene exhibited a predominance of missense mutations, whereas ARID1A displayed a wide variety of mutation types. Nevertheless, approximately 40% of ARID1A mutations were identified as nonsense or frame-shift mutations, which contribute to the decay of mRNA and the loss of ARID1A protein function. In fact, reduced expression levels of PIK3CA and ARID1A proteins were observed in mutated luminal tumors of PIK3CA and ARID1A, respectively (Suppl Fig. 1). As our investigation revealed significant enrichment in ARID1A-PIK3CA mutational co-occurrence exclusively in the luminal subtype, our research aimed to analyze this specific BC subtype.

Subsequently, we investigated the potential correlation between coexisting ARID1A-PIK3CA mutations and various clinicopathological parameters in luminal tumors, such as disease grade, tumor size, patient age, and overall survival. However, our analysis indicated that none of the aforementioned clinicopathological features exhibited a significant association with coexisting ARID1A-PIK3CA mutations. PIK3CA mutations alone were enriched within grade 3 tumors (Table 1, Suppl Fig. 2).

### Immunity-related signaling pathways are associated with coexistent ARID1A-PIK3CA mutations in luminal tumors

**Figure 3:**
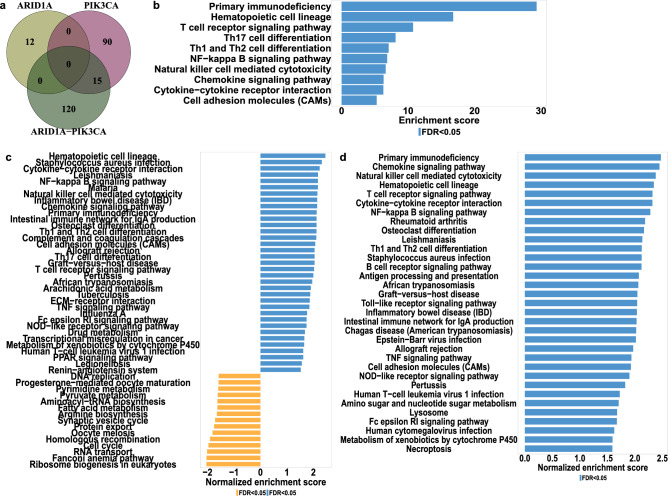
Immunity-related signaling pathways associated with coexistent ARID1A-PIK3CA mutations in luminal tumors. (**a**) Venn diagram of the DEGs sampled at different pairwise comparisons; coexistent ARID1A-PIK3CA mutations (n = 50) versus control samples (n = 899) (dark green), PIK3CA only mutations (n = 780) versus controls (n = 899) (Dark pink), and ARID1A only mutations (n = 51) versus controls (n = 899) (dark olive). (**b**) Over Representation Analysis (ORA) of 120 genes unique to coexistent ARID1A-PIK3CA mutations compared to the control pairwise comparison. (**c**) A gene set enrichment analysis (GSEA) of genes associated with coexistent ARID1A-PIK3CA mutations. (**d**) GSEA analysis of the DEGs sampled at the comparison between coexistent ARID1A-PIK3CA mutations versus PIK3CA only mutation samples.

To obtain an overview of the main gene expression variations in luminal BC patients with coexisting ARID1A-PIK3CA mutations, we conducted several gene expression comparisons (DEGs detected in each comparison are listed in Suppl file 1). Specifically, we identified differentially expressed genes (DEGs) between patients with coexisting ARID1A-PIK3CA mutations and control samples (with no mutations in either ARID1A or in the PIK3CA genes), patients with either ARID1A or PIK3CA mutations alone, and control samples. The Venn diagram in Fig. 3a displays the number of DEGs identified exclusively in each comparison group, as well as the overlapping DEGs. Upon comparing gene expression in ARID1A-PIK3CA mutational co-occurrence versus control samples, we identified a total of 135 DEGs, with 120 genes being unique to this pairwise comparison and not overlapping with other comparison groups. To gain insight into the biological functions of these unique DEGs, we performed an overrepresentation enrichment analysis (ORA) on these genes. Our analysis revealed 13 pathways which were significantly enriched (FDR < 0.05), most of which were immune-related, such as T-cell receptor, B-cell receptor, Th1 and Th2 cell differentiation, Natural killer cell-mediated cytotoxicity, etc. (Fig. 3b). Additionally, to identify potential biological functions associated with ARID1A-PIK3CA mutational co-occurrence tumors, we conducted gene set enrichment analysis (GSEA) and found 49 enrichment pathways, of which 35 were upregulated and 15 downregulated. The majority was related to immune responses (Fig. 3c). Comparing PIK3CA mutated tumors to the control identified 90 unique DEGs (Fig. 3a). GSEA analysis revealed upregulation of 28 pathways and downregulation of 22 of which only nine were immune-related (Suppl Fig. 3). Notably, comparing ARID1A mutated tumors to the control revealed only 12 unique DEGs (FDR < 0.05 with no fold change cutoff). To better comprehend the significance of the coexisting ARID1A-PIK3CA mutations to immune system participation in luminal malignancies, we compared gene expression between patients with coexisting ARID1A-PIK3CA mutations and patients with PIK3CA mutations alone and detected 33 DEGs (FDR < 0.05 with no fold change cutoff) of which 18 are immune related. GSEA analysis revealed upregulation of 33 pathways, 20 of them are immune related (Fig. 3d).

Comparing gene expression between patients with coexisting ARID1A-PIK3CA mutations and patients with ARID1A mutations alone, detected 332 DEGs (312 upregulated genes and 20 downregulated genes). GSEA analysis revealed 41 upregulated pathways, of them 28 are immune related (Suppl Fig. 4).

**Figure 4:**
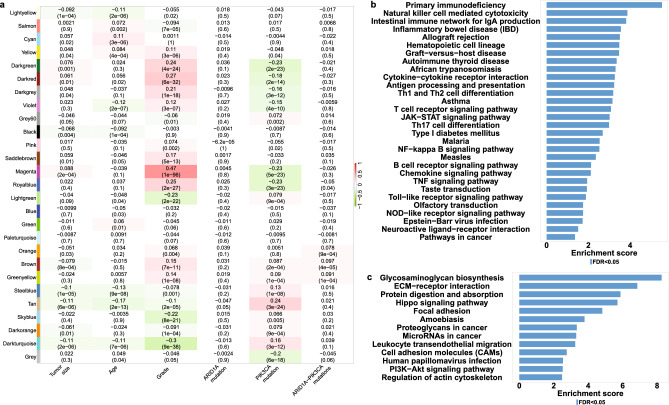
Identification of key modules associated with ARID1A-PIK3CA mutational co-occurrence luminal tumors utilizing weighted gene co-expression network analysis (WGCNA). (**a**) Heatmap of the correlation between consensus module eigengenes and clinical traits. Each column corresponds to a clinical trait, and each row corresponds to a module. Numbers and colors in the table represent correlation coefficients; red marks positive correlation, while green represents negative correlation. The *p* values are shown in parentheses under the correlations. The RNA-seq expression dataset obtained from patients with coexistent ARID1A-PIK3CA mutations (n = 50), PIK3CA only mutations (n = 780) and ARID1A only mutations (n = 51). (b-c) Over Representation Analysis (ORA) for (**b**) ‘brown’ and (**c**) ‘tan’ genes modules.

To confirm our findings with an additional approach, we identified the co-expression network most strongly associated with ARID1A-PIK3CA mutational co-occurrence tumors using Weighted Gene Co-expression Network Analysis (WGCNA). This approach allowed us to identify genes that are highly correlated with specific traits through their co-expression across samples. All luminal tumors were included in the WGCNA analysis. Figure 4a displays the correlations of the module eigengenes with the traits. As expected, most of the gene modules are correlated with pathological features such as the grade. When looking for modules correlated with molecular features, the brown module was found to be the most significantly related to the ARID1A-PIK3CA mutational co-occurrence trait, despite the low correlation (correlation = 0.097, *p* value = 4E-05), followed by the Greenyellow (correlation = 0.091, *p* value = 1E-04) and Orange modules (correlation = 0.078, *p* value = 9E-04). Over-representation analysis (ORA) of the brown module genes showed that most of them were mainly associated with immune pathways, such as primary immunodeficiency, natural killer cell-mediated cytotoxicity, and Th1 and Th2 cell differentiation (Fig. 4b). ORA of the Greenyellow and Orange module genes revealed mainly pathways related to autoimmune diseases and drug metabolism respectively (Suppl. Figure 5).

The tan module was the most correlated to the PIK3CA mutation-only trait (correlation = 0.24, *p* value = 3E-24) (Fig. 4a). Genes included in this module were involved among others in ECM-receptor interaction and protein digestion absorption (Fig. 4c). The immune-related brown module was also significantly associated with PIK3CA mutation-only trait albeit with lower correlation (correlation = 0.087, *p* value = 2E-04) than that of the co-mutation trait. Notably, no module was significantly related to the ARID1A mutation-only trait. To ensure that the presence of an ARID1A mutation in the co-mutated trait is indeed contributing to the association with the immune system, we performed a secondary round of WGCNA analysis. This time, we focused solely on luminal patients who had mutations in the PIK3CA gene. In this analysis, the examined trait is the addition of ARID1A mutation as all patients included here are PIK3CA-mutated.

### Identification of Hub genes associated with ARID1A-PIK3CA mutational co-occurrence luminal tumors

**Figure 5:**
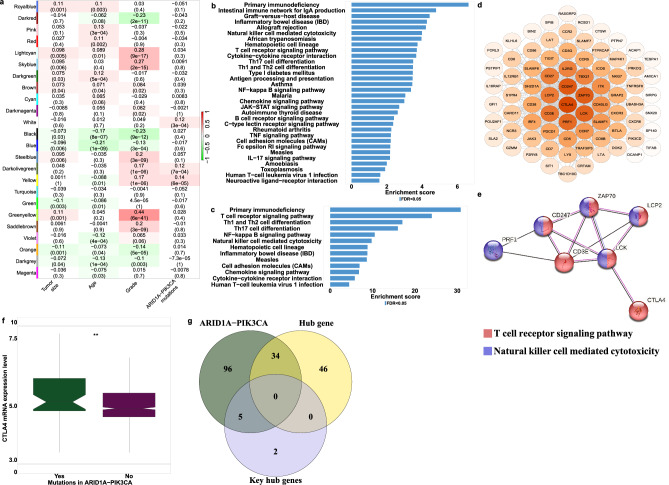
Identification of hub genes associated with immune pathways in ARID1A-PIK3CA mutational co-occurrence luminal tumors. WGCNA analysis of luminal tissues exhibiting PIK3CA mutations (n = 780). (**a**) A module-trait heatmap. (**b**) ORA for the ‘yellow’ module. (**c**) ORA for the top-ranked 5% (with trait gene significance ≥ 0.1 and module membership ≥ 0.8) hub genes in the ‘yellow’ module. (**d**) The protein–protein interaction (PPI) network of hub genes in the ‘yellow’ module is visualized by the Cytoscape software. Colored circles represent the connectivity degree of the hub gene. Darker colors correspond to higher degrees of connectivity. (**e**) The top seven key hub genes with the highest connectivity degrees in a STRING PPI network of the ‘yellow’ module. (**f**) mRNA expression levels of CTL4A were investigated in luminal tumors according to their ARID1A and PIK3CA mutation status. The RNA-seq expression dataset obtained from patients with coexistent ARID1A-PIK3CA mutations (n = 50) and controls (n = 897). The box plots display the expression distribution of CTL4A across the different groups. The line in the middle represents the median value (**p ≤ 0.01, Wilcoxon test). (**g**) Venn diagram showing the overlap between 135 unique DEGs genes of ARID1A-PIK3CA mutational co-occurrence tumors (n = 50) and the seven key hub genes.

PIK3CA is frequently mutated in 40–50% of estrogen receptor-positive (ER +) tumors^43^. Luminal tissues harboring PIK3CA mutations were selected for additional analysis using WGCNA. The correlation between relevant modules and clinical characteristics is demonstrated through a heatmap depicted in Fig. 5a. The Yellow module exhibited the strongest correlation with ARID1A-PIK3CA mutational co-occurrence trait (correlations = 0.14, p value = 6E-05). ORA analysis revealed a significant association with immune signaling pathways (as depicted in Fig. 5b). Subsequently, 87 hub genes representing the top 5% of genes with trait gene significance ≥ 0.1 were identified within the yellow module. The enriched pathways associated with these hub genes were mainly related to the immune system, as shown in Fig. 5c. Using the Cytoscape software and STRING database, a protein–protein interaction (PPI) network was constructed for the hub genes, revealing seven key hub gene with a high degree of connectivity (CTLA4, PRF1, LCK, CD3E, CD247, ZAP70, and LCP2). These genes were found to be significantly associated with immune-related pathways, such as the T-cell receptor signaling pathway and natural killer cell-mediated cytotoxicity, as illustrated in Fig. 5d,e. Notably, CTLA4, one of the hub genes, is known to play a crucial role in immune escape^2^. The expression of CTLA4 was found to be upregulated in ARID1A-PIK3CA mutational co-occurrence tumors compared to the control (as shown in Fig. 5f). Lastly, a Venn-diagram in Fig. 5g revealed an overlap between 135 unique DEGs genes of ARID1A-PIK3CA mutational co-occurrence tumors, 80 hub genes, and the seven key hub genes. These findings collectively suggest that immune-related pathways may contribute to tumorigenesis in patients with ARID1A-PIK3CA mutational co-occurrence tumors.

### The hub genes exhibit strong positive correlations with immune cell infiltration in ARID1A-PIK3CA mutational co-occurrence luminal breast cancer

**Figure 6:**
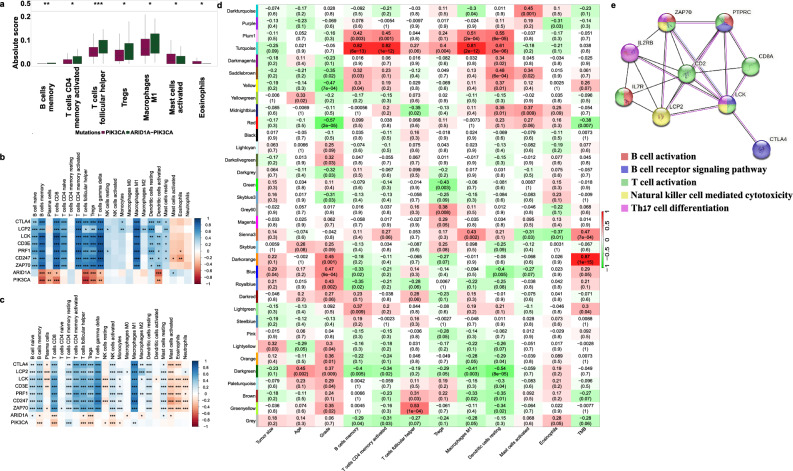
Tumor infiltrated immune cells in ARID1A-PIK3CA mutational co-occurrence luminal tumors. (**a**) Boxplot graphs comparing immune cell infiltration between ARID1A-PIK3CA mutational co-occurrence tumors (n = 50) and PIK3CA-only mutation tumors (n = 780) (*p ≤ 0.05, **p ≤ 0.01, ***p ≤ 0.001, Kruskal–Wallis test). (**b**,**c**) Correlation analysis of the top seven key hub genes, ARID1A, and PIK3CA mRNA expression levels with an absolute score of 22 types of immune cells obtained by the CIBERSORTx algorithm across tumors with (**b**) coexistent ARID1A-PIK3CA mutations (n = 50) or (**c**) PIK3CA-only mutations (n = 780) (*p value ≤ 0.05, **p ≤ 0.01, ***p ≤ 0.001, Pearson correlation test). (**d**) WGCNA on coexistent ARID1A-PIK3CA mutations' luminal tissues (n = 50). The heatmap presents the correlation between consensus module eigengenes and immune cells. (**e**) A PPI network of nine key hub genes of the 'turquoise’ module visualized by STRING.

Due to the link between immune pathways and ARID1A-PIK3CA mutational co-occurrence in luminal tumors, we proceeded to investigate the infiltration of immune cells in the tumor microenvironment by utilizing the CIBERSORTx algorithm. This algorithm employs gene expression profiles to evaluate the abundance of 22 immune cell subsets in the samples^44^. Our analysis revealed a higher proportion of B cell memory, T cells follicular helper, T cells regulatory (Tregs), and Macrophages M1 in ARID1A-PIK3CA mutational co-occurrence luminal tumors compared to the PIK3CA mutation-only group. Conversely, mast cells and eosinophils infiltration were significantly higher in the latter group (Fig. 6a).

We also investigated the immune cells infiltration in ARID1A-PIK3CA mutational co-occurrence luminal tumors compared to both ARID1A mutation-only and control groups (no mutations in these genes at all), and found similar results (Suppl Fig. 6). Additionally, we performed correlation analysis to corroborate the relationship between tumor infiltrated immune cells and the expression of the seven key hub genes (CTLA4, PRF1, LCK, CD3E, CD247, ZAP70, and LCP2) in ARID1A-PIK3CA mutational co-occurrence tumors. Our results indicate a positive correlation between immune cells and these key hub genes, whereas ARID1A and PIK3CA expression exhibited a negative correlation with immune cells (Fig. 6b). A weaker but similar correlation was observed in the PIK3CA mutation-only group (Fig. 6c).

Furthermore, we identified gene sets related to immune infiltration cells in ARID1A-PIK3CA mutational co-occurrence tumors using WGCNA analysis (Fig. 6d). The turquoise module displayed a highly positive correlation with B cells memory, T cells CD4 memory activated, Tregs, Macrophages M1, and resting Dendritic cells. By leveraging the Cytoscape software and STRING database, we identified nine key hub genes in the turquoise module associated with T and B cells' signaling pathways and natural killer-cell-mediated cytotoxicity (Fig. 6e).

### High Tumor Mutation Burden (TMB) in ARID1A-PIK3CA mutational co-occurrence tumors

**Figure 7:**
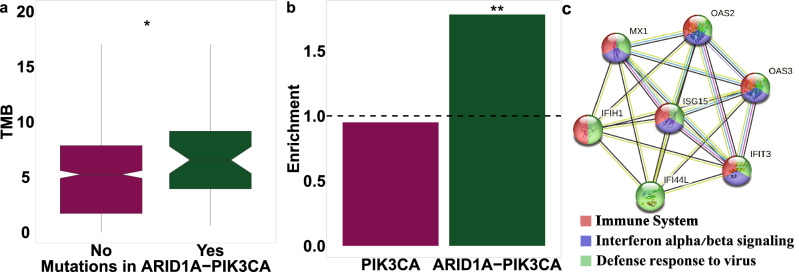
Distribution of Tumor Mutation Burden (TMB) across ARID1A-PIK3CA mutational co-occurrence luminal tumors. (**a**) The box plots display the distribution of the TMB value across ARID1A-PIK3CA mutational co-occurrence tumors (n = 50) compared to PIK3CA mutation only tumors (n = 779). The line in the middle represents the median value (*p ≤ 0.05, Wilcoxon test). (**b**) Bar plot representing the enrichment of tumors with TMB higher than 10 (**p ≤ 0.01, Fisher’s exact test). (**c**) The ‘Sienna3’ module is highly positively correlated with TMB in ARID1A-PIK3CA mutational co-occurrence tumors (n = 50), as demonstrated in Fig. 5d. The hub genes (top-ranked 10%) in the ‘Sienna3’ module are visualized by the STRING PPI network.

Tumor mutational burden (TMB) is a major immune biomarker demonstrated as a useful marker of checkpoint inhibitors' effectiveness and is predictive for immune therapies in advanced BC^45^. Considering the involvement of the immune system in ARID1A-PIK3CA mutational co-occurrence tumors, we evaluated the TMB distribution of the tumors. The median TMB in ARID1A-PIK3CA mutational co-occurrence tumors was higher than in the PIK3CA mutation-only group (Fig. 7a). Estimating the prevalence of patients with TMB above 10 non-synonymous mutations per MB (high TMB) in ARID1A-PIK3CA mutational co-occurrence tumors, we found that high TMB were significantly enriched in ARID1A-PIK3CA mutational co-occurrence tumors compared with PIK3CA mutation-only group (*p* = 0.009, OR = 2.28) (Fig. 7b). Moreover, as shown in Fig. 6d, the Sienna3 module was highly positively correlated with TMB in ARID1A-PIK3CA mutational co-occurrence tumors. Key hub genes of this module are related to the immune system, i.e., defense response to viruses and interferon alpha/beta signaling (Fig. 7c). These results support the potential suitability of luminal ARID1A-PIK3CA mutational co-occurrence tumors for immunotherapy.

## Discussion

Luminal BC represents a highly heterogeneous subgroup in terms of somatic mutations and copy number alterations, which can be characterized by different risks of relapse^4,15,17^. Considering the great genetic heterogeneity of luminal tumors, it seems that classification of a new sub-group in this type of tumor based on genetic alterations could be fundamental, both in predicting the clinical outcome and in therapeutics improvement.

Since ARID1A and PIK3CA mutations are known to cooperate in initiating ovarian and gastric cancer^12,16^, we focused here on luminal tumors with a PIK3CA gene mutation and an additional mutation in ARID1A gene in an attempt to identify a new sub-group of luminal tumors. We analyzed the frequencies and possible involvement in pathogenesis of ARID1A and PIK3CA mutations co-occurrence in luminal tumors. We found that PIK3CA mutations were significantly enriched in luminal tumors, in agreement with other studies^5^. We also characterized, an enriched sub-group of luminal tumors with ARID1A and PIK3CA mutations co-occurrence.

In our analysis of differential gene expression, we observed a relatively high number of DEGs when comparing samples with co-occurring mutations in ARID1A and PIK3CA to the control group. Similarly, we found a substantial number of DEGs when comparing samples with PIK3CA mutations to the control group. However, there were significantly fewer DEGs detected when comparing samples with ARID1A mutations to the control group. We assume, that the presence of various mutation types in the ARID1A gene contributes to increased variability in gene expression within the mutated samples, resulting in a relatively low number of differentially expressed genes. Expanding the sample size would enable a more detailed analysis based on mutation types, potentially reducing sample to sample variability and revealing a greater number of mutation-specific DEGs.

Importantly, we found a strong link between ARID1A and PIK3CA mutation co-occurrence and immune-related pathways, in particular B-cell and T-cell signaling. We also identified seven key hub genes in ARID1A-PIK3CA mutational co-occurrence tumors: CTLA4, PRF1, LCK, CD3E, CD247, ZAP70, and LCP2. As expected, these genes were associated with immune-related pathways. The presence of infiltrated immune cells in these tumors, some of which inhibit or promote disease progression, also supports the idea that immune pathways are involved in this subgroup.

A link between coexistent ARID1A-PIK3CA mutations and immune pathways was previously demonstrated in ovarian cancer^12^. Interestingly, the PIK3CA mutation combines with another gene mutation in immune-related breast tumors. As evidence, PIK3CA and CDH1 mutations act together to induce immune-related invasive lobular carcinoma of the breast^46^.

Our data revealed an association between ARID1A-PIK3CA mutational co-occurrence tumors and immune pathways. A link between the immune response and luminal BC cells where the PI3K pathway is disrupted has already been published, providing evidence that tumor infiltration of CD8-positive lymphocytes is associated with PIK3CA mutations in luminal tumors^47^. The next question would be whether oncogenic ARID1A-only mutations also contribute to immune signaling regulation in this subgroup. We could not answer this question using our data because comparing ARID1A-mutated tumors to the control revealed a very small number of DEGs. However, a recent report shows that ARID1A mutation can enhance the immunogenicity of tumor cells in BC^48^ and were found to be associated with immune activity in gastrointestinal and microsatellite-stable colorectal cancers^49,50^. While we cannot determine with certainty that the ARID1A mutation, in our case, also directly activates or suppresses the immune pathways, we suggest that it contributes to and encourages the general activation of the immune system and increases the activity of the PIK3CA mutation.

Notably, we observed a significant negative correlation between the expression of ARID1A and PIK3CA genes and the presence of infiltrated immune cells. This intriguing correlation remains consistent even though we did not identify any significant alterations in these genes expression when comparing mutated and non-mutated samples. However, both genes exhibited significant decrease of their protein levels. The fact that we don’t see difference in mRNA levels can be explained due to heterogenous mutation types which collectively lead to an overall absence of significant change. Furthermore, the observed negative correlation between gene expression and immune cell infiltration, may result from even small changes in mRNA levels, underscoring the sensitivity of this relationship.

Among luminal tumors, a high proportion of infiltrated immune cells predicted a worse prognosis, suggesting that an efficient immune escape could be an important factor influencing recurrence in luminal BC^2,51^. CTLA4 has an important role in immune escape, as its expression on Tregs could suppress the anti-tumor immune response by inhibiting the proliferation of effector T lymphocytes and was associated with worse disease-free survival and overall-survival^2^. Notably, our work revealed that the key hub gene CTLA4 was associated with ARID1A-PIK3CA mutational co-occurrence luminal tumors, and its expression was higher in these tumors than in the control. Although the exact underlying mechanism remains unclear, considering these cancer-immune interactions in luminal tumors might provide useful information for further development of immunomodulatory combination treatments for luminal tumors.

Nowadays, the efficacy and safety of immunotherapy in luminal tumors have been tested in various ongoing clinical trials^52,53^. For example, a Phase II Trial of Ipilimumab (Anti-CTLA4) and Nivolumab (Anti-PD-1) conducted in unresectable or metastatic metaplastic BC, part of the patients in this trial have luminal tumors^2,54^. It is very important to focus on identifying luminal patients who may benefit from immunotherapy and offer them the appropriate immunotherapy treatment. We hypothesize that the new sub-group of luminal tumors with ARID1A and PIK3CA mutation co-occurrence we identified could benefit from immunotherapy treatment due to the strong association between these tumors and immune-related pathways. Specifically, it is worth testing the efficacy of ICBs, such as CTLA4 inhibitors in this new sub-group, as CTL4A was defined as a key hub gene in these tumors. Moreover, high TMB, a useful marker of checkpoint inhibitors' effectiveness in advanced BC^45^, was significantly enriched in the new sub-group of luminal tumors.

In conclusion, we identified an enriched sub-group of luminal tumors with ARID1A and PIK3CA mutation co-occurrence. Our results reveal different evidence for the involvement of immune-related pathways and tumor-infiltrating immune cells  in these tumors. Particularly, our data raise the hypothesis that ARID1A and PIK3CA mutations' co-occurrence may be predictive for responses to immunotherapy in luminal BC and, if validated, could guide immunotherapy development in this context.

### Supplementary Information

Supplementary Information.

Supplementary Figures.

## Data Availability

The datasets analyzed during the current study are publicly available as normalized expression values and mutational extended data at cBioPortal^23,24^ (https://www.cbioportal.org/study/summary?id=brca_tcga_pub2015%2Cbrca_tcga%2Cbrca_tcga_pub%2Cbrca_tcga_pan_can_atlas_2018%2Cbrca_metabric), and metaGXbreast R package^22^ (https://bioconductor.org/packages/release/data/experiment/html/MetaGxBreast.html). Analyzed data generated during the current study are available from the corresponding author upon request.
