# Supplementary material for: Coexistent ARID1A-PIK3CA mutations are associated with immune-related pathways in luminal breast cancer
Source: Sci Rep. 2023 Nov 27;13:20911. doi: 10.1038/s41598-023-48002-x (PMC10684499; doi:10.1038/s41598-023-48002-x)
Supplement: Supplementary file 2 — Supplementary Figures. [file 41598_2023_48002_MOESM2_ESM.pdf]

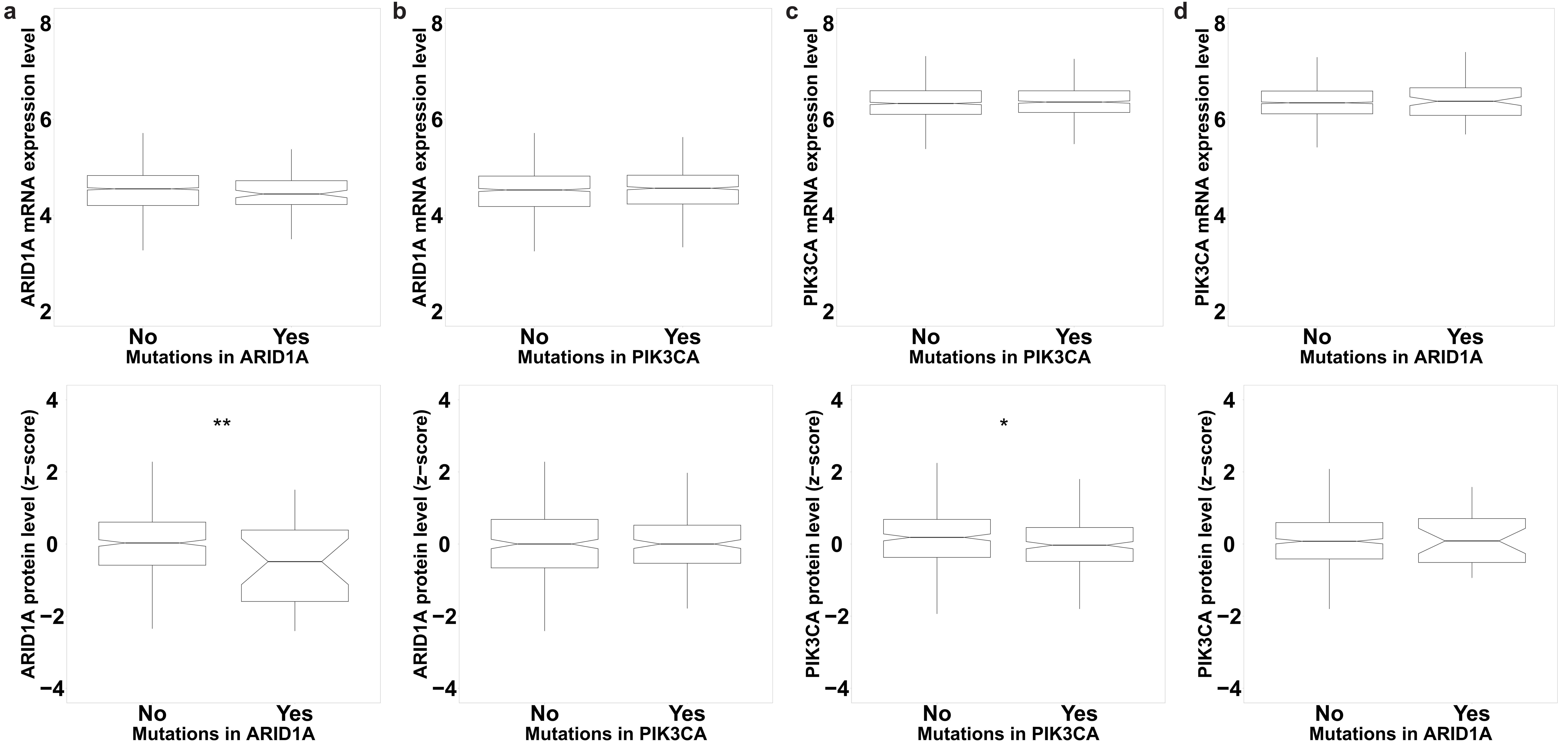

Suppl Fig. 1. **ARID1A and PIK3CA mRNA and protein levels in luminal breast tumors**. Boxplot graphs showing: (a) ARID1A mRNA and protein levels in ARID1A mutated tumors (n=101, n=24, respectively), and in control tumors without ARID1A mutation (n=1679 for mRNA and n=431 for protein expression). (b) ARID1A mRNA and protein levels in PIK3CA mutated tumors (n=830, n=190, respectively) and in control tumors without PIK3CA mutation (n=950 for mRNA and n=265 for protein expression). (c) PIK3CA mRNA and protein levels in PIK3CA mutated tumors (n=830, n=222, respectively) and in control tumors without PIK3CA mutation (n=950 for mRNA and n=304 for protein expression). (d) PIK3CA mRNA and protein levels in ARID1A mutated tumors (n=101, n=30, respectively) and in control tumors without ARID1A mutation (n=1679 for mRNA and n=496 for protein expression). Boxplots representing medians and interquartile ranges of relative ARID1A and PIK3CA mRNA expression (upper panel) and protein z-scores (lower panel). Whiskers represent the minimum and maximum 1.5 interquartile range (*p≤ 0.05, **p≤ 0.01, Wilcoxon test).

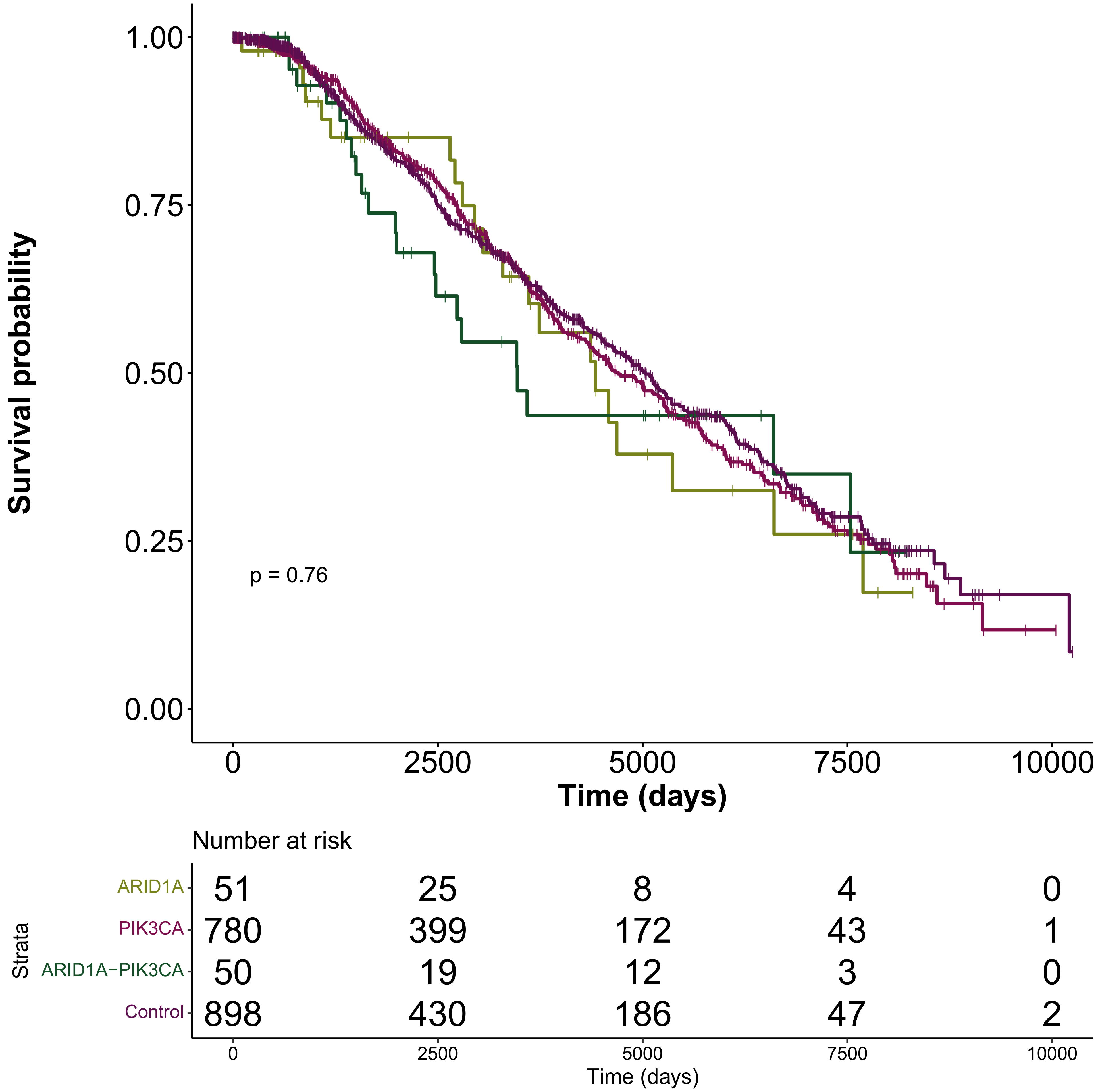

Suppl Fig. 2 **Coexistent ARID1A-PIK3CA mutations not associated with survival in luminal BC**. Survival analysis in luminal breast cancer data set. The patients are divided into four groups according to their ARID1A and/or PIK3CA mutation status: ARID1A-PIK3CA mutational co-occurrence tumors (n=50), ARID1A-only mutation tumors (n=51), PIK3CA-only mutation tumors (n=780) and control tumors (n=898)

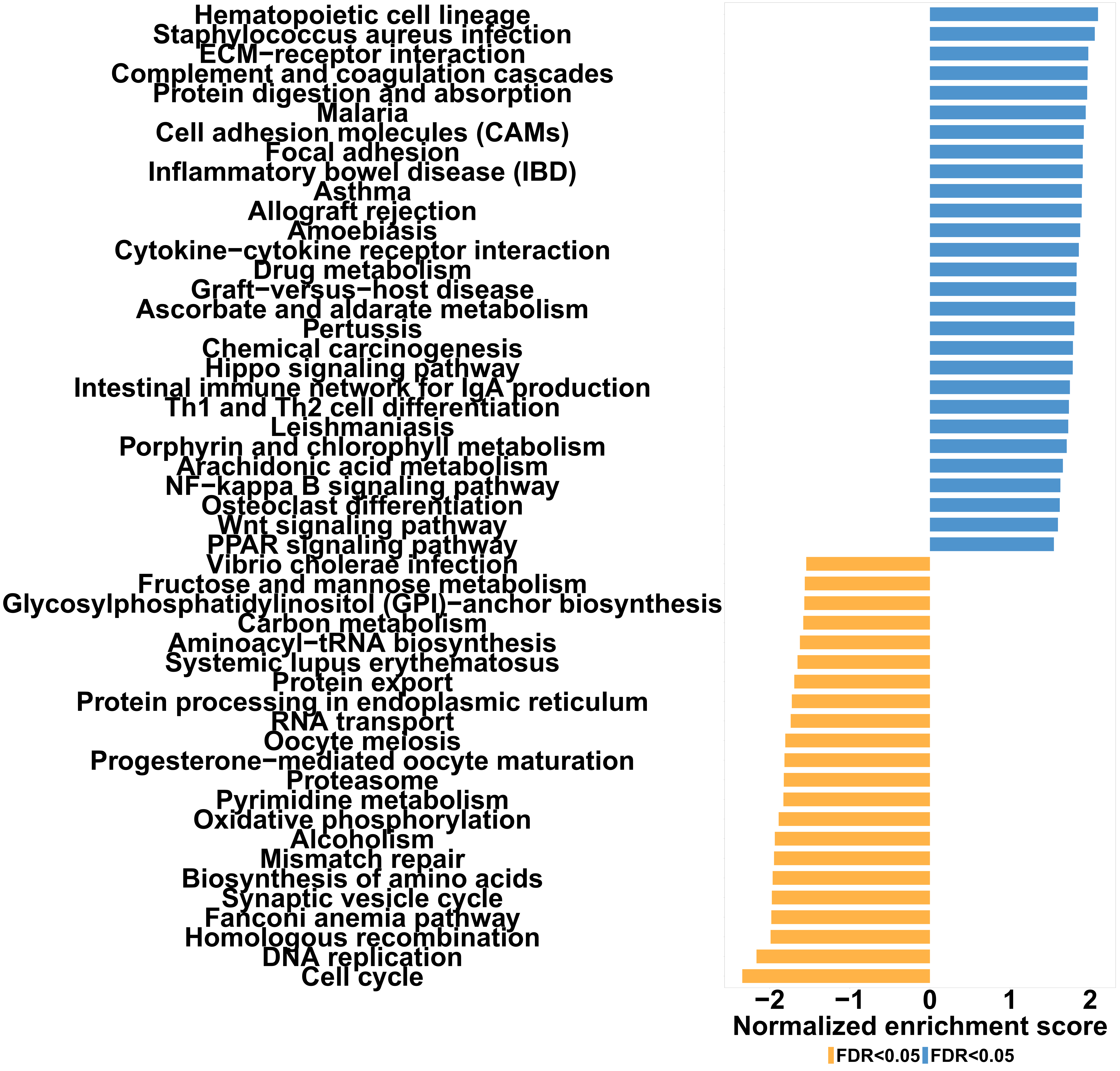

Suppl Fig. 3 Gene set enrichment analysis (GSEA) of DEGs associated with PIK3CA-only mutations.

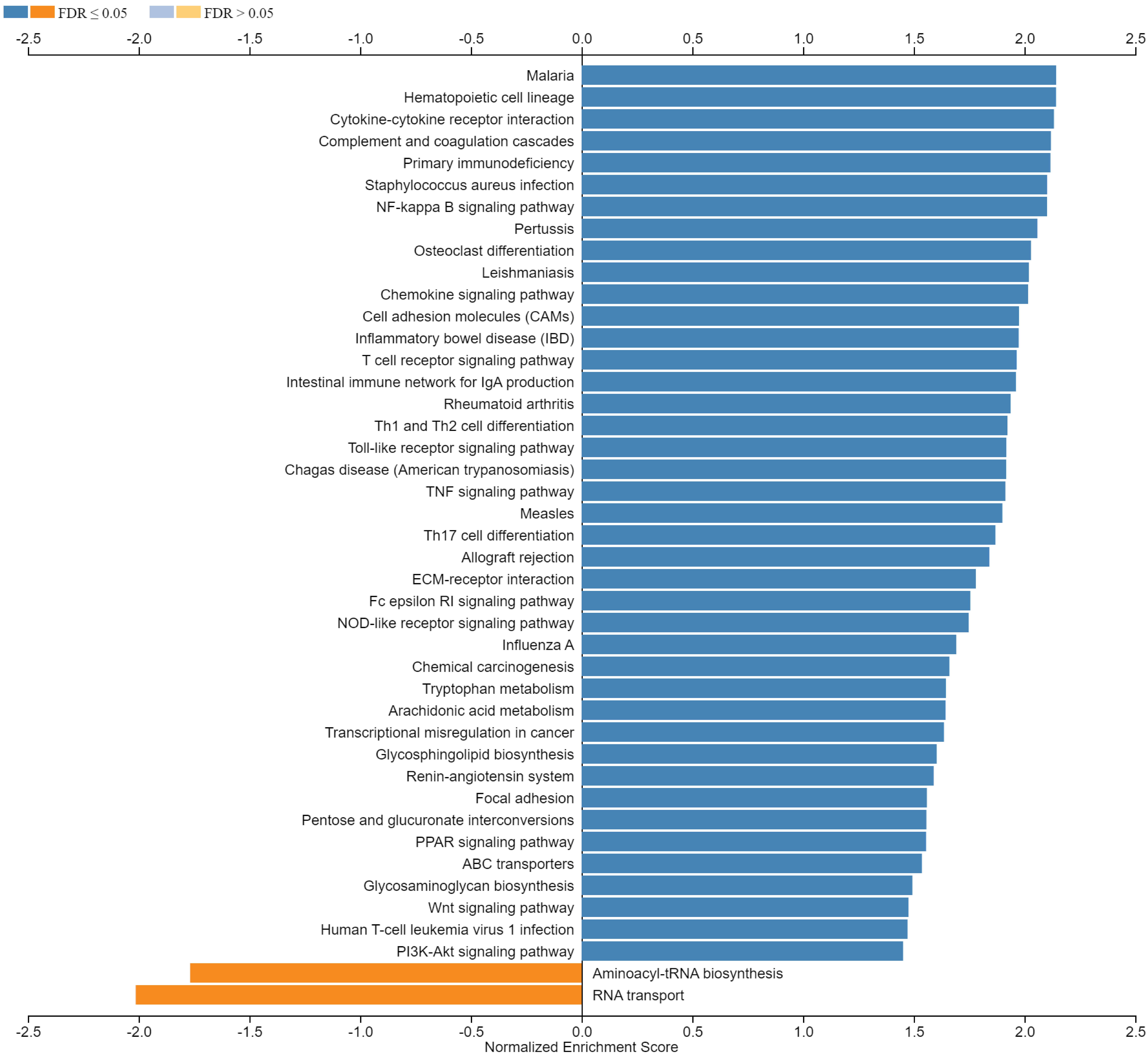

Suppl Fig 4: GSEA analysis of the DEGs sampled at the comparison between coexistent ARID1A-PIK3CA mutations vs. ARID1A only mutation samples.

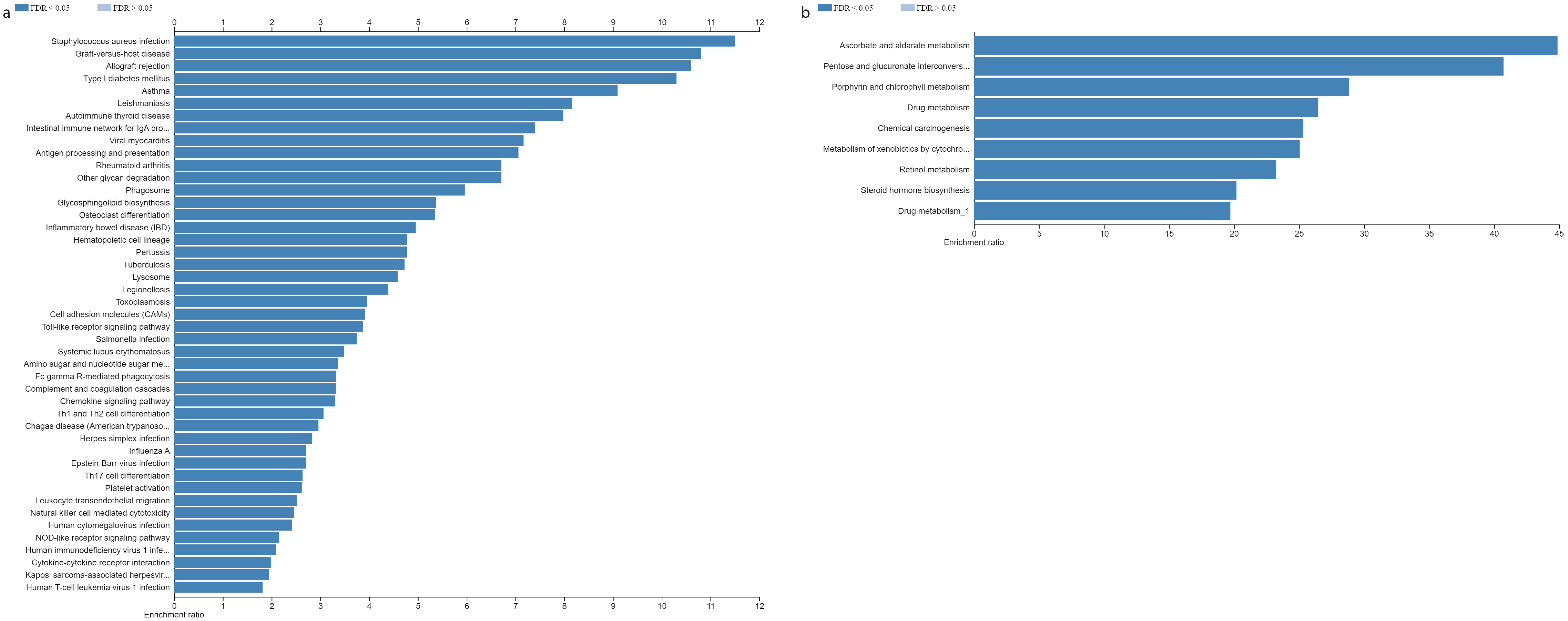

Suppl Fig 5: Over Representation Analysis (ORA) for (a) 'Greenyellow and (b) 'Orange' genes modules.

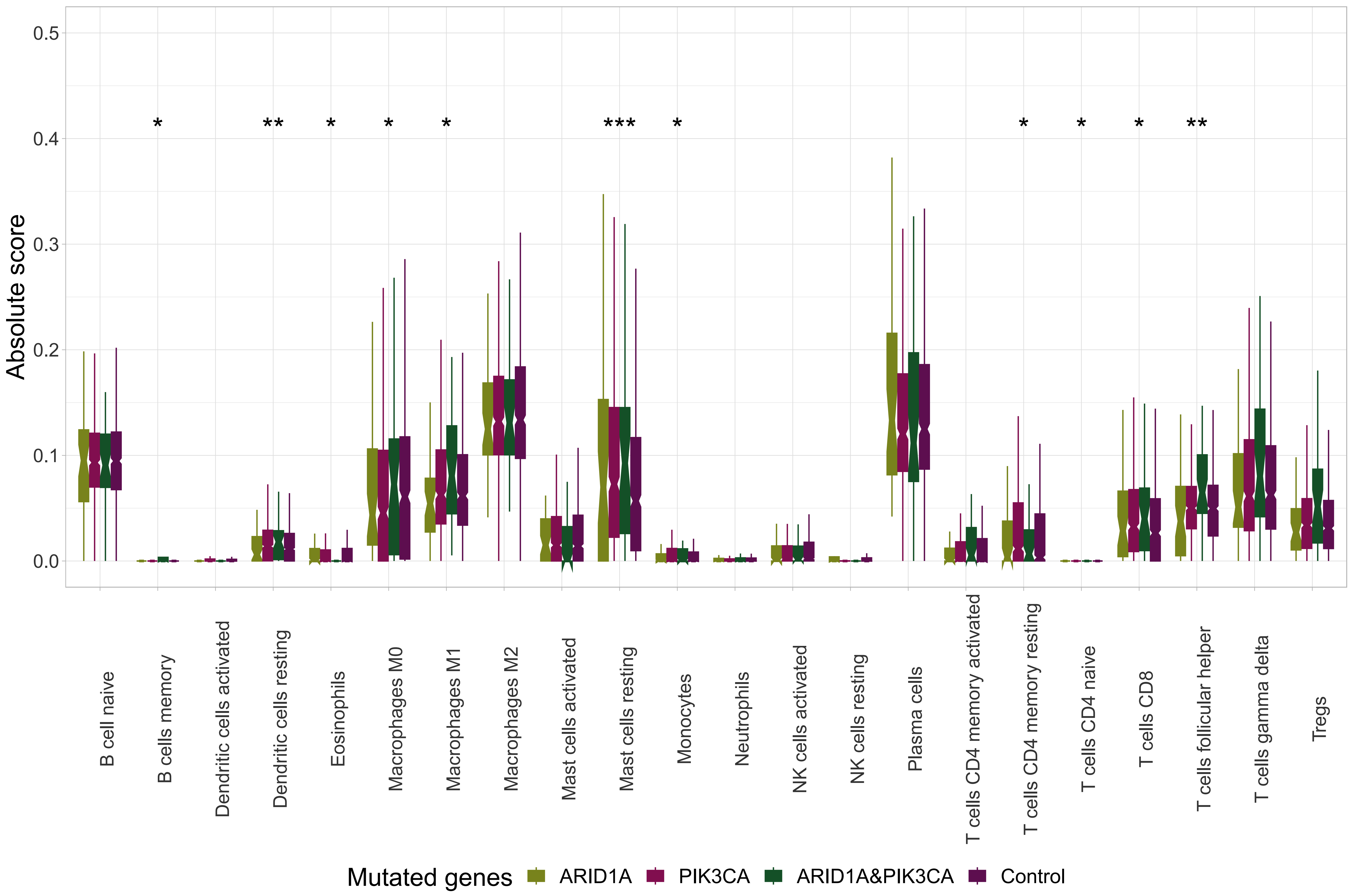

Suppl Fig. 6: Boxplot graphs comparing immune cell infiltration between ARID1A-PIK3CA mutational co-occurrence tumors (n=50) and ARID1A-only mutation tumors (n=51), PIK3CA-only mutation tumors (n=780) and control (n=898) groups. (*p-value ≤ 0.05, **p≤ 0.01, ***p≤0.001, Pearson correlation test).
